# Association between the irrigation-agitation techniques and Periapical Healing of large periapical lesions: a Randomized Controlled Trial

**DOI:** 10.1007/s00784-024-05758-4

**Published:** 2024-06-15

**Authors:** Mehmet Umutcan Doğan, Banu Arıcıoğlu, Taha Emre Köse, Ahter Şanal Çıkman, Merve Çoban Öksüzer

**Affiliations:** 1 Faculty of Dentistry, Department of Endodontics, Recep Tayyip Erdoğan University, Rize, Turkey; 2 Faculty of Dentistry, Department of Endodontics, Istanbul Medeniyet University, İstanbul, Turkey

**Keywords:** Irrigation agitation, LAI, SWEEPS, PUI, Periapical healing, CBCT

## Abstract

**Objectives:**

The aim of this study was to evaluate the effects of manual dynamic activation (MDA), passive ultrasonic irrigation (PUI), and laser-activated irrigation (shock wave-enhanced emission photoacoustic streaming (SWEEPS)) on the periapical healing of large periapical lesions following nonsurgical root canal treatment.

**Materials and methods:**

A total of fifty-six systemically healthy patients with a mandibular single-rooted tooth with periapical lesions of endodontic origin and a periapical index score of 3 or higher were included in the study. Before the treatment procedures, lesion volumes were determined volumetrically using cone–beam computed tomography (CBCT). Patients were randomized into treatment (MDA, PUI, SWEEPS) and control groups (*n* = 14). Root canal treatment and irrigation procedures were performed by a calibrated postgraduate operator and completed at one visit. For routine follow-up, clinical and radiographic evaluations were performed by a blinded evaluator using periapical (PA) radiographs according to Molven’s criteria at 3, 6, and 9 months. At 12 months, lesion volumes were quantified volumetrically using CBCT (ITK-SNAP). The data were statistically analyzed with the Wilcoxon test. The significance level was set at *p* < 0.05.

**Results:**

In all groups, the mean lesion volume after treatment was significantly smaller than the mean volume before treatment (*p* = 0.001). Among the 56 teeth, 11 teeth were ‘totally healed’, and 39 teeth were ‘reduced’ on PA radiographs. No ‘enlargement’ was detected in any group. On CBCT, the lesion volume decreased in the following order: LAI-SWEEPS (86.9%) > PUI (85.4%) > MDA (80.4%) > control (74.5%), with no statistically significant difference (*p* > 0.05).

**Conclusions:**

Despite the limitations of the present study, although a greater percentage of healing was observed in the LAI-SWEEPS and PUI groups, irrigation procedures had no statistically significant effect on the healing of periapical lesions with a single root canal at the 12-month follow-up. On the other hand, the outcome may change in multirooted teeth with curved and complex root canal systems.

**Clinical relevance:**

In the short term and in single-canal teeth, advanced irrigation agitation methods such as laser and ultrasonic did not make a difference in healing other than manual irrigation agitation.

## Introduction

Microorganisms and their products play pivotal roles in the initiation, progression, and establishment of periradicular conditions [1]. The main aim of root canal treatment for teeth with necrotic, contaminated pulp is to treat the infection and prevent future infections. A significant portion of the root canal surface remains untouched during treatment with mechanical instrumentation alone [2]. Therefore, irrigation is crucial for thorough disinfection of the root canal system [3, 4].

Manual dynamic activation (MDA) involves moving a fitted gutta percha master cone in amplitude strokes following canal preparation after canal preparation is completed. This pecking movement aims to increase the effectiveness of disinfection by allowing the solution to contact more surfaces [5]. Passive ultrasonic irrigation (PUI), one of the most widely used activation systems today, creates an acoustic flow by providing hydrodynamic activation and contributes to disinfection by increasing the cavitation effect with the bubbles it produces. When applying this technique, small-diameter tips should not touch the root canal walls and should be used close to the apical region of the canal [6–8]. In recent decades, laser-activated irrigation (LAI) has become popular for debris and smear layer removal and antibacterial efficacy. Shock wave-enhanced emission photoacoustic stream (SWEEPS) is the most recent technology of the Er: YAG laser model used to improve irrigation efficiency. The application of two consecutive laser pulses to the irrigation solution at a certain time ensures that the bubbles created by the first laser beam collapse more quickly and that the photoacoustic shock wave reaches the narrow, inaccessible parts of the root canal [9]. In the SWEEPS technique, it is sufficient to place only the laser tip in the pulp chamber, but in traditional laser activation, canal expansion is needed to reach the laser tip to the root apex. This advantage of the SWEEPS is that it allows minimally invasive endodontic preparation [9, 10]. Yang et al. reported that SWEEPS could emit synchronized laser pulses. In this way, the movement of the irrigation fluid and the bubble collapse rate increase. As a result, the SWEEPS can clear both the main channel and irregularities [10].

Apical periodontitis is a clinical condition characterized by inflammation of periradicular tissues and resorption of mineralized tissues. These manifestations result from the interaction between microbial factors and the host immune response, which is often associated with various systemic diseases [11]. Successful healing of apical periodontitis requires a reduction in the size of the radiolucent area and healing of the bone [12]. The evaluation of periapical pathologies and changes in their volumes with CBCT is more successful than that with 2D radiographs [13]. It is a good guide both to determine different canal variations, size of cysts or endodontic lesions that may affect the treatment plan [14], and to follow the recovery after treatment [15].

In the literature, there are numerous studies on the efficacy of irrigation agitation methods for debris smear removal [16–18], calcium hydroxide removal [19–21] and sealer penetration in dentinal tubules [22–24] under in vitro conditions. However, only clinical studies can clarify their advantages or superiority on periapical healing [25]. Hence this study was conducted to evaluate the efficiency of different irrigation-agitation methods (MDA, PUI, LAI-SWEEPS) on healing rates of large periapical lesions based on volumetric change by CBCT scans during a 1-year follow-up. The null hypothesis was that there was no significant difference in lesion healing between the groups.

## Materials and methods

Local ethics committee approval was obtained from the Ethical Review Committee of the Research Foundation at the Medical Faculty of Recep Tayyip Erdogan University (No: 2023/35) and the study protocol was registered at ClinicalTrials.gov (NCT06204887).

### Sample size calculation

The G Power 3.0.10 (University Kiel, Germany) program was used to calculate the effect size. The effect size was calculated based on chi-square analysis data between the control group and the laser group in the Verma, Yadav study [26]. An effect size of 0.51 Cohen d value was found to be sufficient for significance. With a type 1 error of 0.05, it was determined that at least 56 subjects were required for a total of 4 groups, 14 in each study group, with 95% power.

### Patient recruitment and randomization

Patients were informed about the procedure, and written informed consent was obtained before the commencement of treatment. The study included mandibular single-rooted teeth that were diagnosed with asymptomatic apical periodontitis and had a periapical index (PAI) score of 3 or higher. A total of 97 patients aged 18–65 were evaluated radiographically and clinically for conformity with the inclusion and exclusion criteria. Patients with systemic diseases (diabetes, hypertension, chronic liver disease, coagulation disorders), bone metabolism disease and/or patients using drugs that affect bone metabolism (such as steroids and bisphosphonates) were excluded from the study. Immunosuppressed patients, patients with a history of radiotherapy, pregnant patients, patients with teeth with a mobility of 2 or more (Miller’s mobility index), patients with teeth with a periodontal pocket depth of 5 mm or more, patients with generalized asymptomatic apical periodontitis, patients with teeth with internal and external resorption, and patients with teeth with vertical and horizontal root fractures were not included.

After applying the eligibility criteria, 70 patients were randomly divided into four groups using software (www.random.org) by a blinded researcher who was not otherwise involved in the study according to a standardized procedure. The numbers were placed in dark envelopes and concealed. The envelopes were only opened when the irrigation solution was to be activated. The patients were informed about the study without specifying the group to which they were assigned. All procedures were performed by a single operator with five years of experience (U.D.).

CBCT imaging (Planmeca Romexis, Helsinki, Finland) was requested for patients who met the study criteria before the procedure to obtain information about periapical lesion size, proximity to anatomical landmarks, and anatomical variations of the tooth. CBCT images were acquired with a field of view (FOV) of 5 × 5 cm using ENDO mode, an 85 μm voxel size, 6.3 mA, 90 kV, and 8.7 s.

**Fig. 1:**
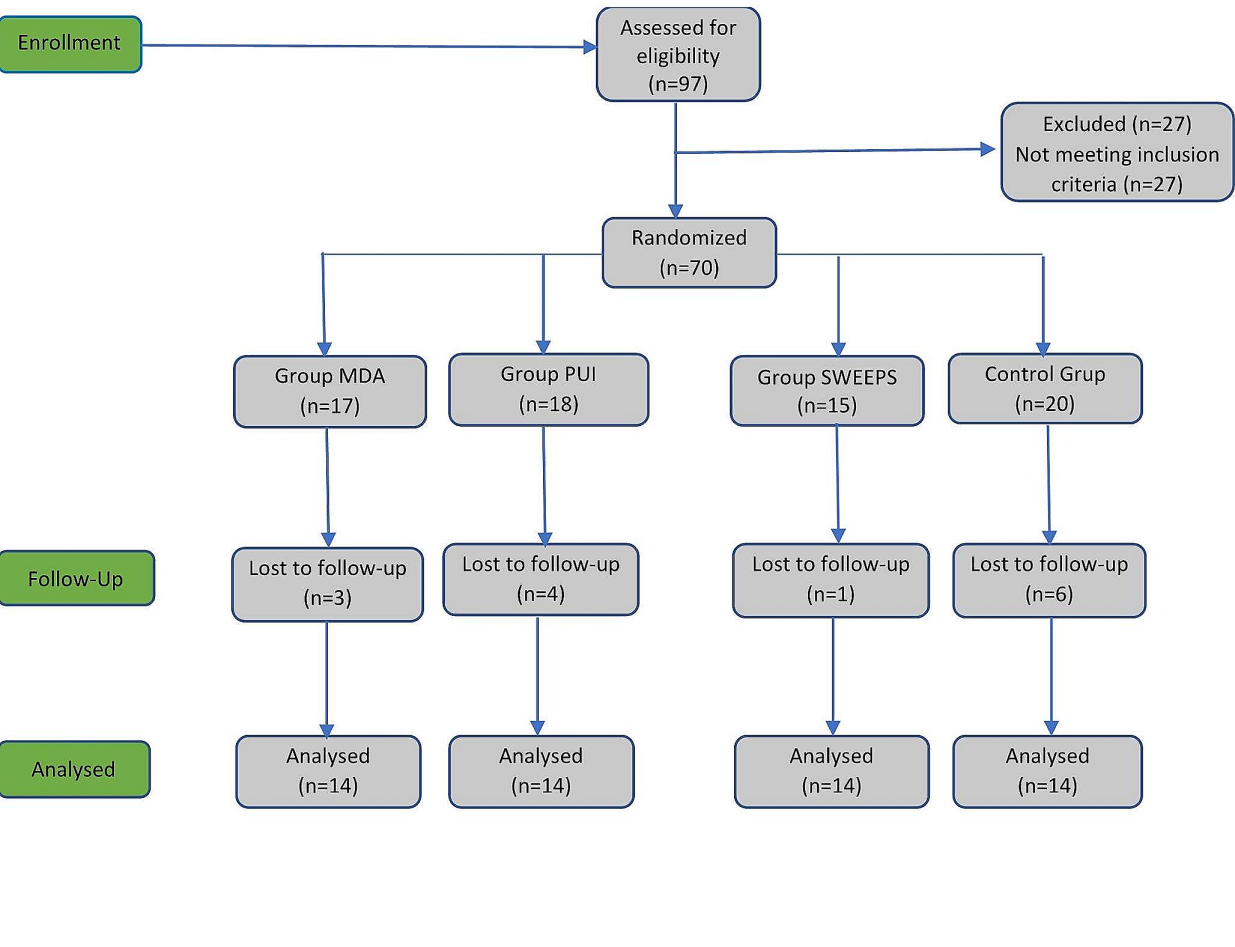
Consolidated Standards of Reporting Trials (CONSORT) flow diagram for patients included in this study

### Clinical procedures

A CONSORT flow diagram outlining the treatment methodology is presented in Fig. 1. After the administration of local anesthesia and the placement of a rubber dam, the access cavity was opened with a sterile diamond round bur under water cooling. Each canal was rinsed with 2 mL of sodium hypochlorite and explored with a size 08 K-file (FKG Dentaire, La Chaux-de-Fonds, Switzerland). The working length was determined using an electronic apex locator, Root ZX mini (J. Morita Co., Tokyo, Japan), to be 0.5 mm shorter than a 0.0 reading. The length was confirmed radiographically. After that, progressively larger K-files were passively introduced into the canal until the operator felt the first one to bind at the WL and the next larger one not to reach that position [27]. The first instrument used to bind the canal was recorded for each canal. The crown-down technique was applied with ProTaper Next rotary files (Dentsply Maillefer, Ballaigues, Switzerland) using a torque-controlled endodontic motor (SybronEndo, Glendora, CA, USA) at 300 rpm/2-5.2 Ncm rotation mode according to the manufacturer’s instructions. The final instrumentation file was set to 3 sizes larger than the first file used [28]. Between each instrument change, the root canal was irrigated with 5 mL of 2.5% NaOCl (Microvem AF, Istanbul, Turkey) for 1 min. After canal preparation was completed, the final irrigation procedure was carried out with the corresponding irrigation method in each group.

### Control Group: conventional syringe irrigation

A 30-gauge side-vented irrigation needle (Kerr Hawe Sa, Bioggio, Switzerland) was used. The needle was inserted into the canal 1 mm shorter than the working length, and the canal was irrigated with up-and-down movements of 1–2 mm amplitude using the same and constant average pressure. The irrigation protocol was performed with 6 ml of 17% EDTA followed by 6 ml of 2.5% NaOCl for 1 min. Between each cycle, 5 ml of distilled water was used to prevent chemical interactions.

### Group 1: manual dynamic activation (MDA)

After the root canal preparation was completed, the final irrigation was started, the main gutta percha cone was positioned 1 mm shorter than the working length, and a 2 mm coronal-apical movement was performed at a speed of 100 strokes/minute for 60 s. The irrigation protocol was performed with 6 ml of 17% EDTA followed by 6 ml of 2.5% NaOCl for 1 min. Between each cycle, 5 ml of distilled water was used to prevent chemical interactions.

### Group 2: Passive Ultrasonic Irrigation (PUI)

In this group, a noncutting ultrasonic tip (IRRI S 21/25; VDW, Munich, Germany) coupled to an ultrasonic device (DTE S6 Led, Guilin Woodpecker Co., Guilin, Guangxi, China) (mode: E, setting: 6) was used according to the manufacturer’s recommendations.

The tip was positioned 2 mm short of the working length without contacting the walls. Continuous irrigation was performed using 2 ml of 17% EDTA followed by 2 ml of 2.5% NaOCl with activation 3 times for 20 s. To prevent chemical interactions between NaOCl and EDTA, 5 ml of distilled water was used between each irrigant. In total, 1 min of irrigation activation was carried out.

### Group 3: laser-activated irrigation (SWEEPS)

In this group, a 2940 nm Er: YAG laser device (Lightwalker, Fotona, Ljubljana, Slovenia) equipped with a handpiece (H14, Fotona) holding an 8.5 mm long and 600 μm diameter tapered fiber tip (SWEEPS 600, Fotona) was used for irrigation activation. The device was set to AutoSWEEPS mode with two ultrashort micropulses (25 µs) continuously varying at 0.3 W, 20 mJ, and 15 Hz. The air and water sprays were turned off.

A 30-gauge side-perforated irrigation needle (Kerr Hawe Sa, Bioggio, Switzerland) was inserted 1 mm shorter than the working length, and the fiber tip was positioned in the center of the access cavity and fixed in this position. Then, 2 ml of 17% EDTA was activated 3 times for 20 s. The same procedure was repeated with 2 ml of 2.5% NaOCl solution by flushing distilled water between each irrigant as described before.

After the final irrigation procedures, the root canals were dried with 25/0.06 paper cones (DiaDent, Heungdeok-gu, Korea), and the cold lateral compaction obturation technique with a root canal sealer (Meta Biomed, Cheongju, Güney, Korea) was used to fill all the canals. Permanent restoration was performed directly with composite resin material (Palfique Estelite, Tokuyama Dental Co., Tokyo, Japan).

### Follow-up procedures

For routine follow-up, 2D radiographs were taken at 3, 6, and 9 months, as well as via intraoral examinations. Radiographic healing in both 2D and 3D at baseline and at a follow-up of 1 year was assessed by a calibrated evaluator who was blinded to the allocation group. The teeth were assessed for reported symptoms, sensitivity to palpation and percussion, mobility and probing depth. The presence of failure (intraoral swelling or sinus tract) was recorded.

### Healing evaluation: lesion area and volume calculation

Twelve months after the root canal treatment, new CBCT images were taken with the same device (Planmeca Promax 3D Classic device (Planmeca Romexis, Helsinki, Finland)) and the same parameters (85 μm voxel size, 6.3 mA, 90 kV, 8.7 with FOV area 5 × 5 cm).

**Fig. 2:**
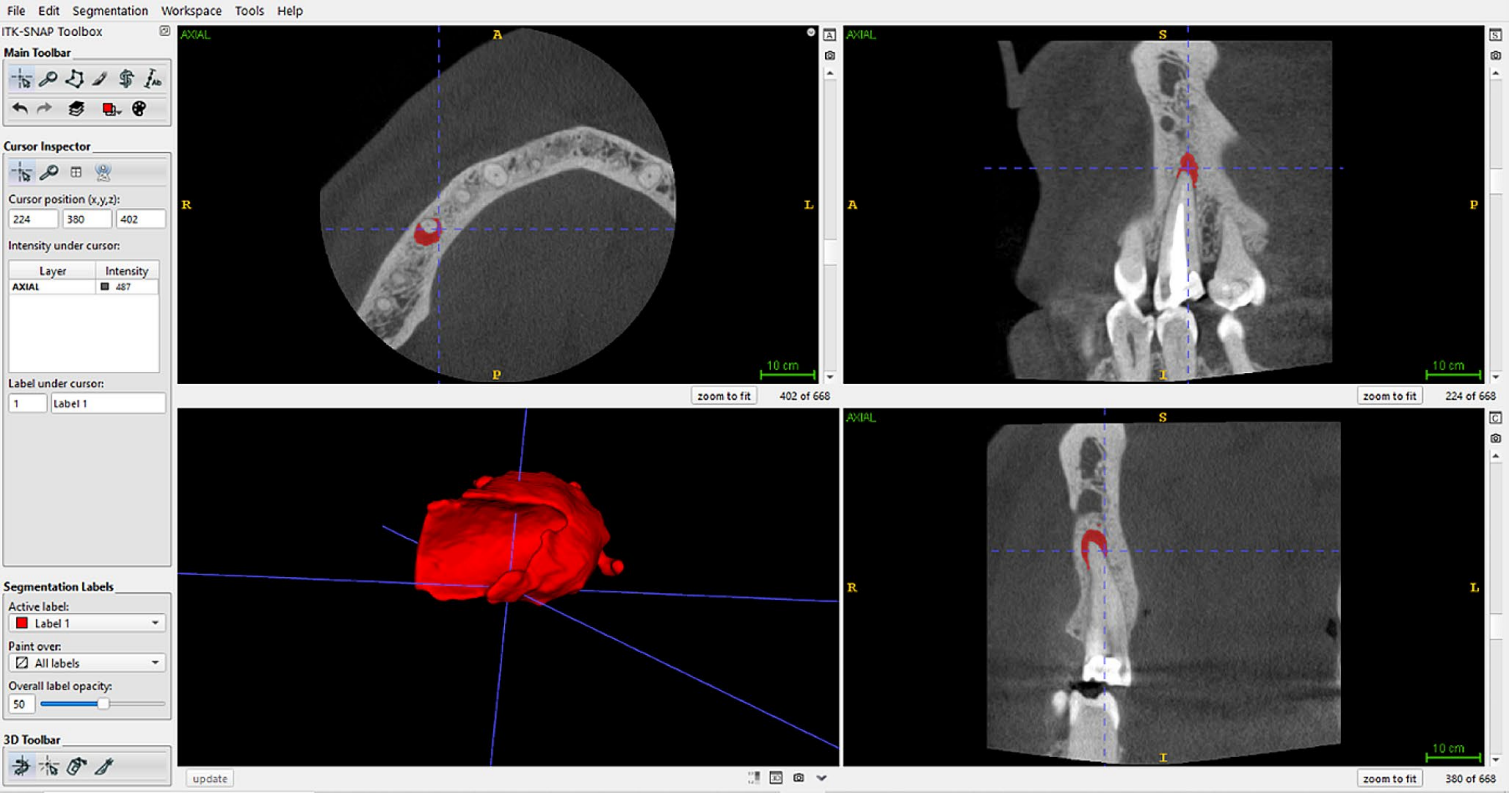
Staining of the patient’s lesion in the ITK SNAP program and obtaining a 3D image

3D lesion volume calculation was performed using ITK SNAP (free software under the GNU General Public License developed by the National Institutes of Health, the US National Institute of Biomedical Imaging and Bioenergy needs, the US National Library of Medicine, the Universities of Pennsylvania and North Carolina, and an independent group of developers) by an oral maxillofacial radiologist with 12 + years of experience **(**Fig. 2**).**

The preoperative and 1-year postoperative CBCT images of the patients were measured following the same steps in the ITK-SNAP program. We used the same techniques used by Schloss et al [15]. First, the captured CBCT images were exported in DICOM file format from the Planmeca Romexis software. The exported images were then opened with ITK-SNAP software. Using the highest resolution allowed by the captured CBCT images (0.09 mm), the slice thickness and slice interval were set to 0.09 mm. The ITK-SNAP program includes a semiautomatic segmentation feature, which was utilized. In this feature, automated spherical fillers, or bubbles, as referred to in the program, were placed according to the grayscale of the lesion. Repeated runs were performed until the entire area of the lesion was filled, with the bubbles placed in the lesion area. After the internal area of the lesion was completely filled, the images were evaluated from axial, sagittal, and coronal sections to correct any possible overfilling or underfilling situations due to artifacts from canal filling materials using a manual marker. The volume of the painted area obtained was calculated in mm^3^ using the program’s feature. The evaluation of the tomography images obtained before and after treatment was conducted at one-month intervals. The images were provided to the evaluators in a randomized manner.

The volume data were compared with the preoperative CBCT measurements for each patient. The volume changes were measured, and the long-term outcomes of the procedures were compared.

### Statistical analysis

Analyses were carried out in the IBM SPSS 25 program. As the first step of the statistical analysis, the normality of the data was checked with the Shapiro–Wilk test. When normality was ensured, the Wilcoxon signed rank test was applied to examine the difference between the means of two dependent groups. ANOVA was used to examine the difference between the means of three or more independent groups. In cases where the data were not normally distributed, the Kruskal‒Wallis test was performed. The post hoc Bonferroni correction was used to determine the group or groups that were significantly different.

Pearson’s chi-square test was applied to examine the relationships between study groups and sex. The Kruskal‒Wallis test was applied to examine the differences between the average ages of the participants in the study groups. Spearman correlations were used to control for the relationships between age and lesion volume measurements obtained at different times and from different study groups. To compare lesion volume measurements according to study group at different measurement times, ANOVA and the Kruskal‒Wallis test were applied. Bonferroni correction were performed to compare the groups. Wilcoxon signed rank tests were used to compare lesion volume measurements according to different measurement times in the study groups.

For comparisons of lesion volume measurements at different measurement times in the study groups, assumptions were checked, and Wilcoxon signed-rank tests were used. Analyses were performed with the IBM SPSS 25 (SPSS Inc., Chicago, IL) program. The Pearson chi-square test was used to examine the relationships between study groups and sex. The Spearman correlation test was used to assess the relationships between lesion volume measurements obtained at different times and study group and age.

## Results

The number of patients lost to follow-up and assessed at the end of 12 months is shown in the CONSORT flow diagram. All patients had no signs/symptoms when assessed clinically during follow-up.

A total of 56 people were included in the study; 22 (39.3%) were women, and 34 (60.7%) were men. The average age of the patients in all groups was homogeneous and similar (*p* > 0.05) (34.5 years) **(**Table 1**)**.

**Table 1:** Demonstration of participants according to age and sex

*Group*	*n*	*Mean Age* *(Range)*	*Female/Male*
*Control*	*14*	*37.073 (21–58)*	*4/10*
*MDA*	*14*	*34.071 (18–60)*	*5/9*
*PUI*	*14*	*38 (20–64)*	*5/9*
*SWEEPS*	*14*	*41.928 (21–63)*	*8/6*
*Total*	*56*	*37.768*	*22/34*

For the volume evaluation between groups, no significant differences were detected in the preoperative measurements. The mean lesion volume at pretreatment was significantly greater than the mean lesion volume at posttreatment in all groups. (*p* < 0.05)

The categorical outcomes of healing regarding the volume changes according to group are presented in Table 2. Among the 56 teeth, 11 (20%) were ‘totally healed’, and 39 (67%) were ‘reduced’, while 6 (11%) were ‘unchanged’ in terms of periapical lesions. No ‘enlargement’ was detected in any group.

For the volume evaluation between groups, no significant differences were detected in the preop measurements (*p* > 0.05), whereas there were significant differences between the postop values in all groups (*p* < 0.005).

There was a statistically significant difference between pretreatment and posttreatment lesion volume measurements in all groups (*p* < 0.05) **(**Table 3**).**

**Table 2:** Lesion volume changes after treatment

Groups (*n* = 4)	Totally healed	Reduced	Unchanged	Enlarged	Total
MDA	3	9	2	0	14
PUI	1	13	0	0	14
SWEEPS	4	9	1	0	14
Control	3	8	3	0	14
Total	11	39	6	0	56

**Table 3:** Lesion volume measurements before and after treatment

Groups	Time	Mean	Standard Deviation	Rank Mean	Test Statistic	*p*
MDA	Preop.	193.936	29.441	8.00	-3.233	0.001*
	Postop.	41.727	66.473	1.00		
PUI	Preop.	158.343	40.275	7.50	-3.296	0.001*
	Postop.	22.813	15.93	0.00		
SWEEPS	Preop.	168.371	37.904	7.50	-3.296	0.001*
	Postop.	23.173	45.055	0.00		
Control	Preop.	172.2	41.707	7.50	-3.296	0.001*
	Postop.	49.646	76.794	0.00		

*p < 0.05

**Table 4:** Comparison of lesion volume change percentages between groups

	Groups	Mean	Standard Deviation	Rank Mean	Test Statistic	*p*
Dimensional Change (%)	MDA	**80.429**	28.294	29.25	2.855**	0.414
	PUI	**85.429**	8.635	23.00		
	SWEEPS	**86.929**	23.332	33.29		
	Control	**74.571**	36.515	28.46		

*p < 0.05 and **Kruskal‒Wallis test

When healing was categorized according to the reduction of the lesion (%) according to group, the SWEEPS and PIPS groups had the highest percentages (87% SWEEPS, 85% PUI), and the control group had the lowest percentage (75%). In the MDA group, the reduction in lesion size was 80%. However, there was no significant difference between the groups (*p* > 0.05) (Table 4).

*A post-hoc power analysis was performed using G Power 3.0.10 (University Kiel, Germany) software. The analysis was based on the findings presented in* Table 4, *yielding a power of 19.8% with an effect size of 0.199 and a total sample size of 56.*

## Discussion

Irrigation activation systems claim improved irrigant transfer, debridement, and removal of smear layer or biofilm. There are several studies in the literature evaluating debris removal [17, 18], antimicrobial [29–32] and effective activation efficiency of activation methods [4, 33, 34]. Many in vitro and tooth model studies support these claims. However, only clinical studies can clarify their advantages or superiority over conventional irrigation method [25]. Hence this study was conducted to show the effectiveness of different irrigation-agitation systemson healing rates of large periapical lesions based on volumetric measurements using CBCT imaging.

Currently, CBCT is widely utilized in studies for various purposes, including assessing dental volume [35], uncovering root canal morphology [36, 37], determining lesion size [14], and serving as one of the most effective diagnostic tools for evaluating regenerated tissue [38–41]. A strong correlation has been demonstrated between CBCT-based predictions and histologic evidence, suggesting that CBCT is an effective noninvasive diagnostic tool for periapical lesions [19, 20, 42–45]. *Schloss et al.* [15] *compared the results of endodontic microsurgery using 2D and 3D imaging methods. CBCT imaging allowed periapical lesion healing to be evaluated more clearly than periapical films. Since the buccolingual width of the lesion is not evaluated in the 2D image, healing or non-healing results may not be interpreted correctly. On the other hand, volume measurement can be perform using 3D imaging, which allows the lesion to be evaluated in all point.* For this reason, in this study, the preoperative and postoperative volumes of periapical lesions were measured with CBCT, and the 1-year outcomes were compared.

The study’s treatment procedure was standardized as much as possible. Standardizing anatomic differences such as root canal curvatures in multirooted teeth and difficulty achieving healthy working lengths and evaluation are more difficult than in single-rooted teeth and may cause errors. Therefore, in this study, teeth with straight and single root canals were used.

The follow-up period for teeth with apical periodontitis varies among studies. The 1-year [45] follow-up period was chosen for most studies [46, 47]. In parallel, Çalışkan et al. [48] reported that the healing of a tooth with a large cyst-like lesion and a wide apex occurred within the first year. Considering the studies, the follow-up period was determined to be 1 year in this study.

*In current study, patients with significant predictive factors of persistent apical disease were exluded. Cases of apical periodontitis should exhibit an asymptomatic repair process with no radiographic abnormalities in the periradicular tissues* [49]. *Even if root canal treatment procedures reach adequate standards, complete healing of the bone or reduction in the size of the apical radiolucency may not occur in all roots* [50].

*Extensive research shows a clear correlation between root canal treatment failure and systemic disease evidenced by the higher rate of postoperative radiolucent periapical lesions among patients with systemic disorder* [51–53]. *These systemic conditions can reduce periapical healing by disrupting bone turnover and fibroblast function* [54] *or may affectting the microvasculature, leading to reduced oxygen and nutrient supply to periapical tissues* [55]. *Therefore, during the tissue repair process, systemic factors such as genetic polymorphism, age, nutrition, stress, hormone levels, vitamin intake, hydration status and diabetes, cardiovascular diseases, osteoporosis and smoking habits should taken into account for the success rates of root canal treatment and healing process.*

According to the volumetric changes, a total of 50 teeth were completely healed or reduced in size, for a success rate of 89.2%. 14 of them (28%) were in the PUI group, 13 in the SWEEPS group (26%), 12 in the MDA group (24%)11 and 11 in the control group (22%). The ‘unchanged’ lesions were mostly observed in the control group (conventional syringe). No increase in lesion volume was observed in any group. The decreases were as follows: SWEEPS (86.9%) > PUI (85.4%) > MDA (80.4%) > conventional syringe (74.5%). Although the success rates in the PUI and LAI-SWEEPS groups were greater than those in the MDA and control groups, which was indicative of better healing, the difference was not statistically significant. According to the comparisons, we failed to reject the null hypothesis. In view of these findings, irrigation with or without *agitation* was crucial for the healing of periapical lesions. However, irrigation agitation techniques did not improve the healing of periapical lesions in single and straight-rooted teeth. *Similarly with our findings, in their systemic analysis, Silva et al. found no evidence supporting the superiority of PUI over non-activated irrigation in terms of enhancing periapical healing and bacterial disinfection in clinical practice* [56]. *In contrast, Susila et al., reported that irrigation activation methods are effective in reducing postoperative pain and cleaning the canals. Because mechanical movement of the irrigation fluid delivered more irrigant to the apical area and debris removal was more effective. However, they stated that there is no enough evidence to support their superiority in delivering irrigant to the apical area* [25].

*According to the study of Liang et al., in single-root teeth, root canal treatments with or without additional ultrasonic activation of irrigation contributed equally to periapical healing. Gender, master cone size, length or density of the canal filling did not affect the results* [57]. *The results obtained in Tang et al.‘s study were that ultrasonic activation and conventional irrigation did not make a significant difference in healing after 19 months of follow-up.* [58].

*The differences in the results of the studies could be for many reasons as as presence of pre-operative symptoms, pre-operative lesion size, Master Apical File size, presence/absence of flare-ups during treatment. Although there are reports supporting the superiority of activation devices in delivering irrigation up to study length, most activation devices have not been clinically evaluated and no controlled clinical studies have been performed to confirm this.*

Due to the lack of clinical studies examining the effect of activation methods on the healing of apical periodontitis, the effectiveness of activation methods was compared with that of in vitro studies. In a study comparing the ER: YAG (2940 nm) laser and PUI methods, there was no significant difference in debris removal efficiency between the two groups [17]. Another study revealed that no significant difference between an ER: YAG (2940 nm) laser and the PUI method in terms of debris removal efficiency [59]. In a study investigating the antimicrobial efficacy of these two methods, no significant difference was found between laser activation and PUI methods [31]. These results are consistent with the statistical data from the Er: YAG (2940 nm) laser and PUI groups in this study.

Zhu X et al. [60] examined the effects of Er: YAG laser and conventional syringe irrigation on smear layer removal and antibacterial effects and reported no significant difference between the two methods. In another study, the smear removal efficiencies of Er: YAG (2940 nm) lasers and conventional syringes were compared in vitro. No significant difference was found between the laser and conventional syringe groups [61].

In a systematic review, Caputa et al. [62] concluded that ultrasonic activation was not superior to conventional syringing for periapical tissue healing. The differences between these results may be attributed to several reasons, including statistical power analysis and sample size, enhanced activation not resulting in statistically significantly superior healing, and variations in irrigation protocols used in the studies.

In contrast to the results of the present study, in a study comparing the debris removal efficiency of LAI and conventional syringes, the LAI method was found to be superior [63]. In another in vitro study investigating the removal of smear layers and debris from the curved mesiobuccal canals of mandibular teeth, the LAI and PUI methods were found to be superior to the conventional methods. The exclusion of curved canals in this study may have caused a limitation in demonstrating the superiority of LAI-SWEEPS and PUI technology over the other methods. *Similarly, Vatanpour et al. reported in their study, there was no significant difference in smear removal efficiency between SWEEPS and PUI, but both methods removed the smear layer better than traditional syringe irrigation* [64]. *In another study, SWEEPS provided higher cleaning efficiency compared to PUI* [65]. *According to the study conducted in vitro design on mandibular single teeth, Saber et al. reported that final irrigation activation with MDA resulted in better removal of the smear layer than PUI or syringe irrigation* [66]. *Differences between the results of the studies may be due to different reasons such as tooth selection, tooth anatomy and morphology, apical preparation diameter, and final irrigation protocol.*

The current study was a prospective, randomized controlled trial with an optimal sample size. The follow-up period of the study was 1 year, which may have limited our ability to obtain definitive evidence of lesion changes in the long term. In addition, in multirooted teeth with curved and complex root canal anatomy, irrigation techniques may provide significant clinical benefits and improve outcomes.

*Although a pre-study power analysis was conducted using data from a previous study* [26], *one of the study’s limitations was the low post-hoc power analysis result of 19.8%. The reasons for the low power include an insufficient sample size, a low expected effect size, and high alpha margins of error. Given the high standard deviations in the study results and the absence of a normal distribution, it is possible that the samples have not been adequately standardized due to the clinical nature of the study. Therefore, it is challenging to ascertain whether the result is due to inadequate power or the absence of a significant effect. It is recommended that future studies be conducted with larger sample sizes to address these limitations.*

Another limitation concerns the 100% reliability of the CBCT. Although CBCT has high reliability, the margin of error can reach 18% [67, 68]. In one study, evaluations of the size of periapical lesions were made with volume data of 20% or more [57]. CBCT evaluation in combination with histologic evidence may be a better diagnostic way to determine the true nature of the healing process in tissues. However, it is unethical to procure postoperative healed tissue from patients to compare the histologic findings with those of CBCT scans [19, 42]. Further research with larger sample sizes would be beneficial for analyzing the efficacy of irrigation *agitation* techniques on the morphology of multirooted and curved root canals.

## Conclusion

Irrigation with or without agitation was crucial for the healing of periapical lesions. However, irrigation–agitation techniques did not improve the healing of periapical lesions in single and straight-rooted teeth. Although the LAI-SWEEPS and PUI irrigation activation methods resulted in a greater rate of apical periodontitis healing, there was no significant difference compared to that in the other groups at the 1-year follow-up.

Further studies could be beneficial for analyzing the efficacy of irrigation-agitation techniques in multirooted teeth with curved and complex root canal morphologies. Long-term randomized clinical trials with large sample sizes are necessary to allow more reliable comparisons between results.

## Data Availability

No datasets were generated or analysed during the current study.
